# No maternal health without perinatal mental health: a framework for standardized indicators

**DOI:** 10.3389/fgwh.2026.1860039

**Published:** 2026-07-02

**Authors:** Francesca Palestra, Tarun Dua, Shanon McNab, Elly Layton, Elissa C. Kennedy, Caroline S. E. Homer, Jane Fisher, Simone Honikman, Zelee Hill, Allisyn C. Moran, Neerja Chowdhary, Tatiana Taylor Salisbury

**Affiliations:** 1 Department of Sexual, Reproductive, Maternal, Child, Adolescent Health and Ageing, World Health Organization (WHO), Geneva, Switzerland; 2 Department of Mental Health, Brain Health and Substance Use, World Health Organization (WHO), Geneva, Switzerland; 3 MOMENTUM Country and Global Leadership, Jhpiego, Washington, District of Columbia, WA, United States; 4 Women's, Children's and Adolescent Health Program, Burnet Institute, Melbourne, VIC, Australia; 5 Public Health and Preventive Medicine, Monash University, Melbourne, VIC, Australia; 6 Perinatal Mental Health Project, Department of Psychiatry and Mental Health, University of Cape Town, Cape Town, South Africa; 7 Institute for Global Health, University College London, London, United Kingdom; 8 Centre for Global Mental Health, King's College London, London, United Kingdom

**Keywords:** depression, health monitoring, indicators, maternal health, mental health conditions, newborn health, perinatal mental health, pregnancy

## Abstract

Perinatal mental health (PMH), the psychological wellbeing of women during pregnancy and up to one year postpartum, is increasingly recognized as a critical determinant of maternal and child health. Despite substantial evidence demonstrating the prevalence and consequences of perinatal mental health conditions, global monitoring systems rarely include standardized indicators to measure these conditions or the services addressing them. This gap limits the ability of countries to track progress, allocate resources, and design effective interventions. In this perspective article, we present the development of a proposed framework of indicators for global monitoring of perinatal mental health. The framework was informed by a scoping review of existing indicators, expert consultations, and two rounds of interest-holder surveys coordinated with the World Health Organization (WHO) and the Mother and Newborn Information for Tracking Outcomes and Results (MoNITOR) advisory group. Indicators were prioritized according to relevance, feasibility, validity, and their potential to support global advocacy and accountability. The resulting framework proposes six perinatal mental health indicators for future testing: three primary indicators, namely the existence of a national PMH policy or plan, prevalence of depressive symptoms, and screening coverage during the perinatal period; and three secondary indicators related to health system capacity and service provision. While implementing standardized monitoring will require strengthening health systems, workforce capacity, and data collection mechanisms, integrating these indicators into existing platforms such as routine health information systems and national surveys offers a feasible pathway forward. Systematic monitoring of perinatal mental health is essential for advancing maternal and newborn health agendas globally. Establishing a core set of indicators can support policy prioritization, guide resource allocation, and accelerate progress toward more comprehensive maternal health care that includes mental health.

## Introduction

Perinatal mental health is increasingly recognized as a fundamental component of maternal health, yet it remains one of the most neglected aspects of global health monitoring. Perinatal mental health (PMH) refers to the psychological wellbeing of individuals during pregnancy and up to one year postpartum (1, 2). During this period, women may experience a range of mental health conditions, including depression, anxiety disorders, psychosis, and substance use disorders (3–5).

The burden of perinatal mental health conditions is substantial. Globally, it is estimated that between 10% and 20% of women experience depression during pregnancy or the postpartum period, with higher prevalence reported in low- and middle-income countries (LMICs) (6, 7). Multiple factors contribute to vulnerability during this period, including socioeconomic stressors, gender inequality, family or domestic violence, cultural expectations, and biological changes (8).

Poor perinatal mental health has significant consequences not only for mothers but also for the babies. Maternal depression is associated with adverse outcomes, including an increased risk of maternal suicide, as well as negative birth outcomes such as preterm birth and low birth weight. These factors, in turn, contribute to long-term developmental and behavioral difficulties in children (9–11). These impacts highlight the intergenerational consequences of unaddressed mental health conditions during pregnancy and early motherhood.

Despite growing awareness and evidence, perinatal mental health remains largely absent from global monitoring frameworks. No standardized indicators are routinely tracked across countries within health information systems or major international surveys.Existing indicators are fragmented and often limited to specific national contexts, primarily in high-income settings (12–14). Additionally, available indicators focus narrowly on diagnosis or treatment rather than on broader health system capacity, prevention, or service access. There is a need for more comprehensive and integrated approaches to perinatal mental health monitoring that address the complexity of healthcare systems and policies across countries, ensuring integration across levels of care and within maternal, newborn and child health services (6, 15).

In this perspective, we argue that establishing a standardized set of global indicators is essential to elevate perinatal mental health within maternal and newborn health agendas. To address this gap, we present the development of a proposed set of indicators informed by global consultations and outline the opportunities and challenges associated with implementing these indicators within existing health monitoring systems.

## Advancing global monitoring of perinatal mental health

A multi-stage, four-phase methodology was employed to develop global perinatal mental health indicators, guided by a WHO expert working group and informed by a prior scoping review. This process included iterative consultations and two rounds of anonymized online surveys with diverse international interest holders, followed by expert review and application of predefined inclusion and exclusion criteria to ensure the indicators were valid, feasible, and globally relevant.

## A theory of change for global perinatal mental health monitoring

Recent global efforts have begun to address the measurement gap in perinatal mental health. A Global Perinatal Mental Health Theory of Change (ToC) proposed a strategic framework for strengthening monitoring and program development (16). The ToC highlights pathways through which improved monitoring can contribute to better maternal and newborn outcomes.adopts a social-ecological perspective encompassing five domains: the individual, interpersonal relationships, community context, service delivery systems, and policy environments. Monitoring indicators play a critical role in linking policy commitments, service delivery, and population health outcomes.

The ToC emphasizes that monitoring systems must capture both health outcomes and the enabling health system structures that support care.

## Evidence from existing indicators

To identify candidate indicators for global monitoring, a scoping review, updated in 2024, examined existing measures used in perinatal mental health monitoring systems worldwide (15). The review considered the indicators that: (1) aligned with the Global PMH ToC and landscape analysis definition of perinatal mental health (during pregnancy and up to one year after childbirth); (2) had a definition, including how they could be measured; and (3) were published from January 2000 to October 2024.

The scoping review methodology exclude indicators related to risk factors for perinatal mental health (PMH) or those that did not directly measure PMH outcomes. Additional indicators assessing general mental health, but not specific to the perinatal period, were also excluded, as these are well described in the WHO Mental Health Atlas (17). Nine indicators were identified in the scoping review across two main domains of the ToC: individual-level outcomes and service delivery characteristics. Indicators included measures such as the prevalence of postpartum depression, screening coverage for mental health conditions during the perinatal period, and the proportion of healthcare providers trained in mental health care.

However, the review revealed important limitations. Most indicators focused on measuring depression after childbirth, while fewer addressed screening, prevention, or health system capacity. In addition, many indicators were designed for specific national contexts, limiting their comparability across countries.

These findings reinforced the need for a concise set of globally relevant indicators that could be integrated into existing monitoring platforms.

## A multi-stage consultation process

Building on the scoping review, a multi-phase consultation process was conducted between January 2024 and April 2025 to identify and prioritize potential indicators for global monitoring (Table 1). This process was guided by the WHO Mother and Newborn Information for Tracking Outcomes and Results (MoNITOR) advisory group and a working group of global experts, involving global, regional, and national experts in maternal health, mental health, and monitoring and evaluation.

**Table 1:** Phases to develop global PMH indicators.

Phase 1	Phase 2	Phase 3	Phase 4
Establishment of WHO expert group on PMH monitoring and discussion on identified indicators of the scoping review	First survey with WHO expert group on PMH monitoring	Second survey with WHO expert group on PMH monitoring and additional suggested stakeholders from global, regional and country-level	Consultation with WHO expert group on PMH monitoring and the WHO MoNITOR advisory group and development of global PMH indicators

In the first survey, members of the expert working group prioritized indicators identified in the scoping review. Indicators related to service delivery, particularly screening coverage, access to services, and provider training, were ranked most highly.

A second survey involving a broader group of interest holders further refined these priorities. Respondents ranked indicators according to importance for global monitoring and suggested additional measures related to policy commitment and financing.

Consultations with the WHO MoNITOR advisory group subsequently assessed the feasibility of measuring these indicators in diverse settings. Particular attention was given to ensuring that selected indicators could be integrated into existing health information systems or national surveys.

Inclusion and exclusion criteria were established by the WHO MoNITOR advisory group to ensure the selection of indicators that met predefined quality standards and objectives. Indicators were included if they demonstrated validity, reliability, feasibility, and potential impact for advocacy purposes. They also needed to be relevant to the assessment of perinatal mental health conditions and be able to measure progress towards improving maternal and newborn health outcomes for global monitoring purposes. Indicators were excluded if they lacked empirical evidence, reflected only risk factors for developing PMH conditions rather than indicators of the conditions themselves, were overly complex or resource-intensive or were specific to a particular context and not generalizable to a broader population.

This work is based on experts' perspectives, including the WHO MoNITOR advisory group on monitoring and measurement, and it is not original research. Therefore, we did not seek approval from the WHO ERC for ethical clearance.

Written consent from members of the WHO expert group, following WHO protocols for expert group participation, which include declarations of interest and confidentiality agreements.

## Proposed global indicators for perinatal mental health

Based on process, a set of primary and secondary indicators was proposed to guide global monitoring efforts (see Tables 2, 3).

**Table 2:** Primary indicators for global monitoring.

Monitoring domain	Indicator	Numerator	Denominator	Current preferred data source	Other data sources
Output	Proportion of countries with national policy/plan for perinatal mental health in SRMNCAH or in the mental health policy/strategy	Number of countries with national policy for perinatal mental health in SRMNCAH policy/strategy or in the mental health policy/strategy	Total number of countries	Policy review or WHO Policy survey	
Outcome	Proportion of women with depressive symptoms^1^ in the perinatal period^2^	Number of women identified with depressive symptoms within a given time period	Total number of women who gave birth within a given time period	Household survey	RHIS*
Outcome	Proportion of women screened for symptoms of mental health conditions in the perinatal period ^2^	Number of women who were screened for mental health conditions within a given time period	Total number of women who gave birth within a given time period	Household surveys	RHIS*

1 Data from mental health screening, as may occur during RHIS data collection, will generate a prevalence for symptoms of a disorder. The robustness of data are dependent on the psychometric and construct validity of the screening tool for the setting and thus need to be interpreted with the appropriate degree of caution. Data from clinical assessment or diagnostic testing will be more accurate. However, these types of tests are seldom conducted at scale. Thus, interpretation of prevalence data from these sources need to take into account the generalisability of the population sampled.

2 Perinatal period: during pregnancy and up to 1 year after pregnancy.

* RHIS, Routine health information system.

**Table 3:** Secondary indicators for global monitoring.

Monitoring domain	Indicator	Numerator	Denominator	Current preferred data source	Other data sources
Output	Proportion of government allocated expenditure to perinatal mental health	Total government allocated expenditure to perinatal mental health	Total government allocated expenditure on health^1^		
	Proportion of in-service health care providers trained to provide mental healthcare services^2^ in perinatal period	Number of in-service health care providers trained to provide mental healthcare services in the perinatal period	Total number of in-service health care providers providing services in the perinatal period	Health facility assessment	NHAs*
Outcome	Proportion of women with perinatal mental health condition linked to psychosocial support OR mental health services	Number of women with perinatal mental health condition linked to psychosocial support OR mental health services	Total number of women with perinatal mental health condition	Household survey	RHIS

1 Relatively crude indicator.

2 Mental healthcare services include a variety of interventions, and it is not limited to screening of mental health conditions. Please refer to the WHO Guide for integration of perinatal mental health in maternal and child health services. Geneva: World Health Organization; 2022.

* NHAs, National health assessments.

### Primary indicators

Primary indicators were selected for their immediate relevance, feasibility, and potential to support advocacy and accountability (Table 2)

1. Existence of a national policy or plan addressing perinatal mental health within broader maternal or mental health strategies.
2. Prevalence of depressive symptoms during the perinatal period.
3. Proportion of women screened for symptoms of mental health conditions during the perinatal period.

Together, these indicators capture three critical dimensions of PMH: policy commitment, population-level burden, and service delivery.

The proposed primary indicators for perinatal mental health monitoring capture key aspects of maternal psychological wellbeing during pregnancy and up to one year postpartum. They were selected for their relevance in assessing policy commitment, population burden, and health system capacity related to perinatal mental health. These indicators serve as core metrics for monitoring progress and informing policies to improve maternal and newborn outcomes. They include measures such as the proportion of countries with a national policy or plan addressing perinatal mental health, the prevalence of antenatal and postnatal depressive symptoms, and screening coverage for symptoms of perinatal mental health conditions.

### Secondary indicators

Secondary indicators were identified to support the continued expansion of monitoring systems as data capacity improves (Table 3). These include:

4. Government expenditure allocated to perinatal mental health
5. Proportion of health providers trained to deliver perinatal mental health services
6. Proportion of women with identified perinatal mental health conditions receiving psychosocial or clinical care

These indicators reflect broader health system capacity and service coverage, which are essential for translating policy commitments into effective care.

Secondary indicators are proposed to guide continuing development of monitoring systems as capacity and data availability improve. They are critical to ensure the sustainability of perinatal mental health services. Without these indicators, it would not be possible to effectively address the PMH burden, strengthen the comprehensiveness and equity of monitoring efforts, or respond to emerging priorities.Together, these indicators support ongoing progress in monitoring and improving perinatal mental health globally.

## Future directions for measurement and implementation

The proposed indicators identify aspects and impact of care which may be more easily integrated into existing data collection, monitoring and evaluation processes. However, further work is needed to deepen our understanding of perinatal mental health needs and care provision. These include the integration of indicators into existing health information systems, improving the ability of the health workforce to adequately and appropriately identify and address mental health challenges, and identifying and addressing barriers to the delivery of perinatal mental health care and support.

## Integrating indicators into existing data systems

A key challenge in implementing standardized monitoring systems is ensuring feasible data collection across diverse health systems (18, 19). In many LMICs, national health information systems remain limited in their capacity to capture mental health indicators. Integrating PMH indicators into existing platforms, such as routine national health information systems or major international surveys such as Demographic and Health Surveys (DHS) or Multiple Indicator Cluster Surveys (MICS), offers a pragmatic solution (20). Household surveys can provide population-level estimates of prevalence, while facility-based systems can track screening coverage and service delivery (21–23). Linking data from household surveys with health facility assessments may further improve measurement accuracy by combining information on care-seeking behavior with service availability and quality (22).

## Strengthening health workforce capacity

Task-sharing approaches, which enable non-specialist health workers to deliver mental health interventions, have shown promising results in several LMIC settings (24, 25). Training and supportive supervision programs should therefore include competencies and assessment frameworks related to screening, counseling, referral, and culturally sensitive care. Clinical services should be strengthened through expanded access to specialist care and enhanced training opportunities for frontline healthcare providers (26). Investing in workforce development will be essential for translating monitoring indicators into meaningful improvements in care.

## Addressing implementation challenges

Despite the potential benefits of standardized monitoring, several challenges remain. Resource constraints, particularly in low-income settings, may limit the ability to collect and report new indicators. Technical issues such as data quality, reporting mechanisms, and interoperability between information systems may further complicate implementation (22). Addressing these challenges will require sustained investment in health systems strengthening, technical support, and international collaboration (23).

## Discussion

Perinatal mental health is a critical yet underrecognized component of maternal and newborn health. The indicatorproposed in this Perspective represents an important step toward addressing this gap.

Translating these indicators into practice will require a phased and context-sensitive approach. Countries can begin by aligning the proposed indicators with existing maternal and newborn health monitoring frameworks, such as routine health information systems and national surveys (e.g., DHS or MICS), to minimize additional reporting burden. Initial steps include identifying a national focal point for perinatal mental health, reviewing existing data systems to map where indicators can be integrated, and selecting validated screening tools appropriate to the local context, as recommended in WHO guidance (2, 18). Pilot implementation in selected regions or facilities can help assess feasibility, data quality, and resource needs before national scale-up. Strengthening workforce capacity through training on screening, referral, and data reporting is essential, alongside incorporating indicators into clinical registers and digital health platforms where possible. Lessons from integrating mental health into primary care and maternal services in LMICs demonstrate that task-sharing approaches and linkage of household and facility data can support both service delivery and monitoring (21, 22, 24). Together, these steps provide a practical pathway for embedding perinatal mental health indicators within existing health systems while progressively strengthening data quality and use.

Importantly, monitoring should not be viewed as an end in itself but as a tool for improving care. Over time, expanding the monitoring framework to include additional indicators related to treatment coverage, quality of care, and infant outcomes could further strengthen global accountability.

Ultimately, advancing perinatal mental health monitoring requires coordinated action across sectors. Governments, international organizations, researchers, and civil society must work together to integrate mental health into maternal health strategies and ensure that women receive comprehensive care during pregnancy and the postpartum period. Recognizing that there can be no maternal health without mental health, establishing global monitoring systems for perinatal mental health represents a critical step toward improving outcomes for women, newborns, and families worldwide.

## Data Availability

The original contributions presented in the study are included in the article/Supplementary Material, further inquiries can be directed to the corresponding author.
